# Sensitive detection of fluorescence in western blotting by merging images

**DOI:** 10.1371/journal.pone.0191532

**Published:** 2018-01-19

**Authors:** Yukari Kondo, Shinichiro Higa, Takeshi Iwasaki, Tomoya Matsumoto, Kazumitsu Maehara, Akihito Harada, Yoshihiro Baba, Masatoshi Fujita, Yasuyuki Ohkawa

**Affiliations:** 1 Division of Transcriptomics, Medical Institute of Bioregulation, Kyushu University, Fukuoka, Japan; 2 Department of Cellular Biochemistry, Graduate School of Pharmaceutical Sciences, Kyushu University, Fukuoka, Japan; 3 Sales Department, Microscope Division, Keyence, Osaka, Japan; 4 Division of Immunology and Genome Biology, Medical Institute of Bioregulation, Kyushu University, Fukuoka, Japan; University of Minnesota Medical Center, UNITED STATES

## Abstract

The western blotting technique is widely used to analyze protein expression levels and protein molecular weight. The chemiluminescence method is mainly used for detection due to its high sensitivity and ease of manipulation, but it is unsuitable for detailed analyses because it cannot be used to detect multiple proteins simultaneously. Recently, more attention has been paid to the fluorescence detection method because it is more quantitative and is suitable for the detection of multiple proteins simultaneously. However, fluorescence detection can be limited by poor image resolution and low detection sensitivity. Here, we describe a method to detect fluorescence in western blots using fluorescence microscopy to obtain high-resolution images. In this method, filters and fluorescent dyes are optimized to enhance detection sensitivity to a level similar to that of the chemiluminescence method.

## Introduction

Cells are regulated according to the central dogma, which is the flow from genetic information to protein expression. The expression levels of proteins dictate cell fate; for example, the expression levels of certain transcription factors regulate skeletal muscle differentiation [1]. Thus, it is critical to quantify protein expression to understand cellular phenomena or potential at the molecular level. Western blotting is the standard method to quantify protein expression [2]. The chemiluminescence method is generally used to detect proteins because of its high sensitivity and ease of use [3, 4]. However, recent developments in proteomics have focused on technologies to detect the expression of multiple proteins simultaneously, as opposed to the chemiluminescence method that detects single proteins. One method for simultaneous detection of multiple proteins which allows more quantitative analysis is fluorescent western blotting [5–7]. However, the detection sensitivity of this method is lower than that of the chemiluminescence method.

In this study, to improve the resolution and sensitivity of fluorescent western blotting, we focused on the fluorescence microscopy step. The microscope’s CCD camera is capable of capturing high-resolution images, but it has a small visual field. Thus, we captured an image with a high resolution and a wide field by merging multiple fluorescence micrographs. In addition, we successfully increased the detection sensitivity to a level comparable to that of the chemiluminescence method by optimizing the filters and fluorescent dyes.

## Materials and methods

### Cells

The mouse C2C12 myoblast cells (CRL-1772, ATCC) were cultured in Dulbecco’s modified Eagle’s medium (DMEM) containing 20% fetal bovine serum, and were harvested at about 70% confluency. Differentiated cells were transferred to DMEM medium containing 2% (v/v) horse serum and cultured for 24 or 72 hours before analyses.

### Immunoblotting

Purified recombinant GST was denatured in bromophenol blue (BPB)-free 2×sodium dodecylsulphate (SDS) sample buffer at 95°C for 5 min. Cells were washed twice in phosphate buffered saline (PBS), resuspended in BPB-free 2×SDS sample buffer, and denatured at 99°C for 2 min. The samples were separated by SDS-PAGE (12% acrylamide), and transferred to an Immun-Blot® LF PVDF Membrane (BioRad, Hercules, CA, USA). The membrane was blocked with 5% skim milk powder in Tris-buffered saline containing 0.05% Tween 20 (TBST), then incubated with primary antibodies in Hikari Solution A (Nacalai Tesque Inc., Kyoto, Japan), and subsequently incubated with secondary antibodies in TBST. The primary antibodies included rabbit monoclonal anti-GST (EPR4236, Abcam, Cambridge, MA, USA; 1:1000), rabbit monoclonal anti-desmin (Y66, Abcam; 1:1000), rat monoclonal anti-Hsp90 alpha (4H7A5, Kishida, Osaka, Japan; 1:2500) and mouse monoclonal anti-myogenin (F5D, Santa Cruz Biotechnology, Santa Cruz, CA, USA; 1:1000). The secondary antibodies included horseradish peroxidase-conjugated anti-rabbit IgG antibody (GE Healthcare Life Science, Buckinghamshire, UK; 1:5000), CF™488A goat anti-rabbit IgG (H+L) (Biotium, Hayward, CA, USA; 1:5000), Alexa Fluor™ 546 goat anti-rabbit IgG (Thermo Fisher Scientific, Rockford, IL, USA; 1:5000), Alexa Fluor™ 647 goat anti-rabbit IgG (Thermo Fisher Scientific, 1:5000), Alexa Fluor™ plus 488 goat anti-rabbit IgG (Thermo Fisher Scientific, 1:5000), Alexa Fluor™ plus 555 goat anti-rabbit IgG (Thermo Fisher Scientific, 1:5000), Alexa Fluor™ 546 goat anti-mouse IgG (H+L) (Thermo Fisher Scientific, 1:5000), ECL Plex goat-α-rabbit IgG, Cy5 (GE Healthcare Life Science, 1:5000), and Alexa Fluor™ 647 chicken anti-rat IgG (H+L) (Thermo Fisher Scientific, 1:5000). Chemiluminescence was analyzed using Chemi-Lumi One Ultra (Nacalai Tesque Inc.).

### Visualization and data analysis

The membrane was placed on a black acrylic plate and covered with cover glass (85 × 125 mm, No. 1-S, Matsunami Glass, Osaka, Japan). Images were visualized under a fluorescent microscope (BZ-X710, Keyence, Osaka, Japan) with a replaced objective lens (CFI Plan Apochromat λ 4×, Nikon Instruments Inc., Tokyo, Japan) and joined using its software BZ-analyzer (Keyence). The fluorescence microscope was equipped with a motorized stage and a CCD camera with the dynamic range of 14 bit, and excitation light emission was kept at the minimal level (low-photobleach mode). The filters included BZ-X filter GFP (OP-87763, Keyence), BZ-X filter Cy5 (OP-87766, Keyence), BZ-X filter TRITC (OP-87764, Keyence), ET-Narrow Band EGFP to minimize autofluorescence (49020, Chroma Technology, Brattleboro, VT, USA), ET-Cy5 narrow excitation (49009, Chroma), and ET-Gold FISH (49304, Chroma). Images were optimized using Adobe PhotoShop CS6. Quantification was performed using ImageJ and relative integrated density was calculated as follows: (area of GST × mean GST)–(area × mean of average of two control areas).

## Results

### Joining images to capture high-resolution fluorescent western blot image

**Fig 1:**
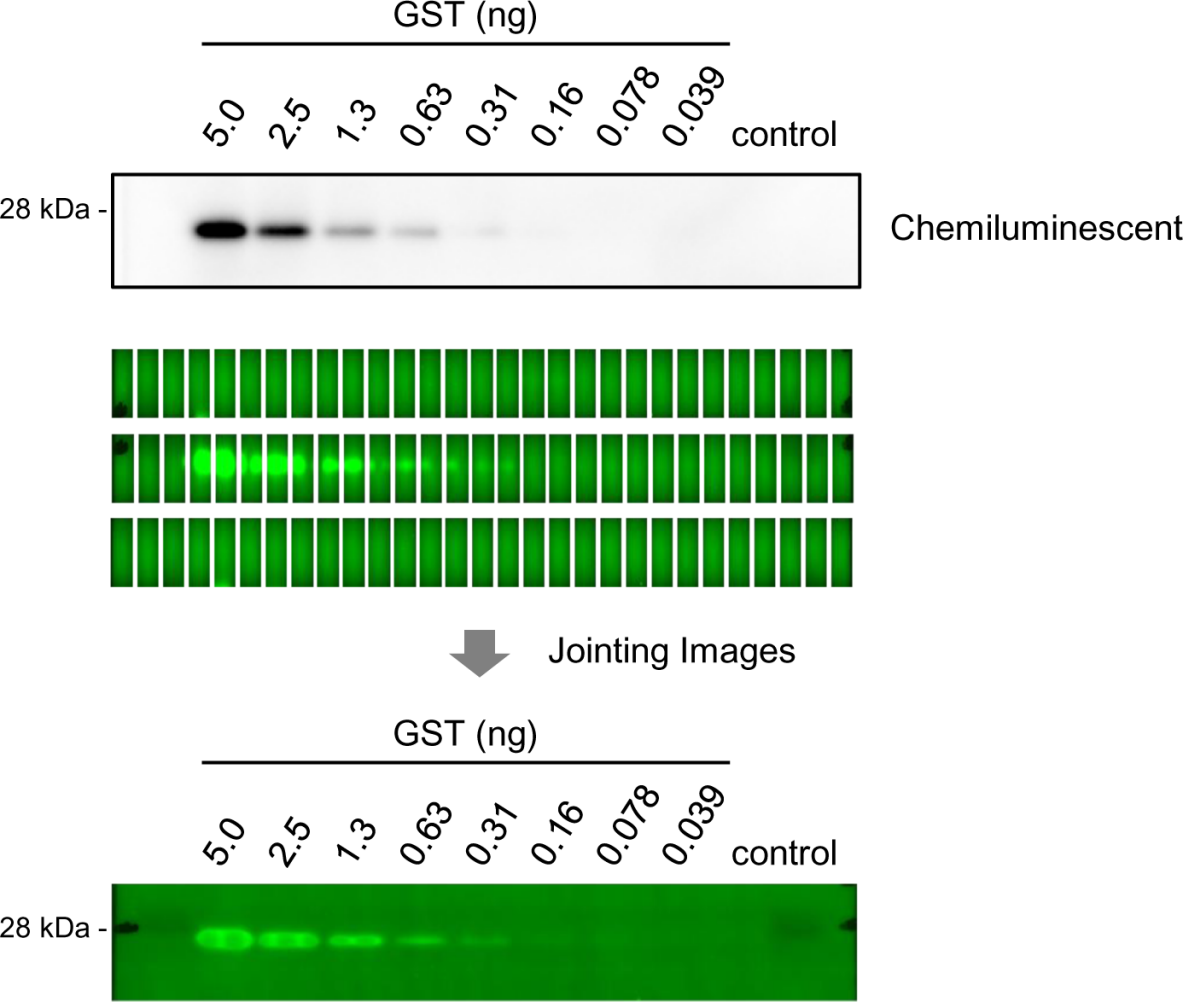
Joined image generated using fluorescence detection method. Dilution series of recombinant GST detected by our fluorescence method and by the chemiluminescence method. In our method, multiple images are captured using fluorescence microscopy, and joined to make a single image with a wide field and high resolution. The joined image has a broad area and high sensitivity comparable to that of the chemiluminescence method.

To perform fluorescent western blotting with high resolution and high detection sensitivity, we captured high-resolution images under a fluorescence microscope and merged those images. Microscopic images (8.8 × 6.6 mm) overlapping by 30% of the image on each side were captured in three rows of 29, and then joined and shade corrected automatically using the BZ-analyzer software. As a result, we successfully captured an image with a wide field, high resolution, a broad area, and sensitivity comparable to that of the chemiluminescence method (Fig 1). Because we used the low-photobleach mode with short-time exposure, fluorescence signal was mostly not repressed. However, the captured image contained noise signals resulting from membrane autofluorescence, leakage of excitation light, nonspecific signals and transmitted scattering light.

### Narrowing transmission wavelength range to enhance detection sensitivity

**Fig 2:**
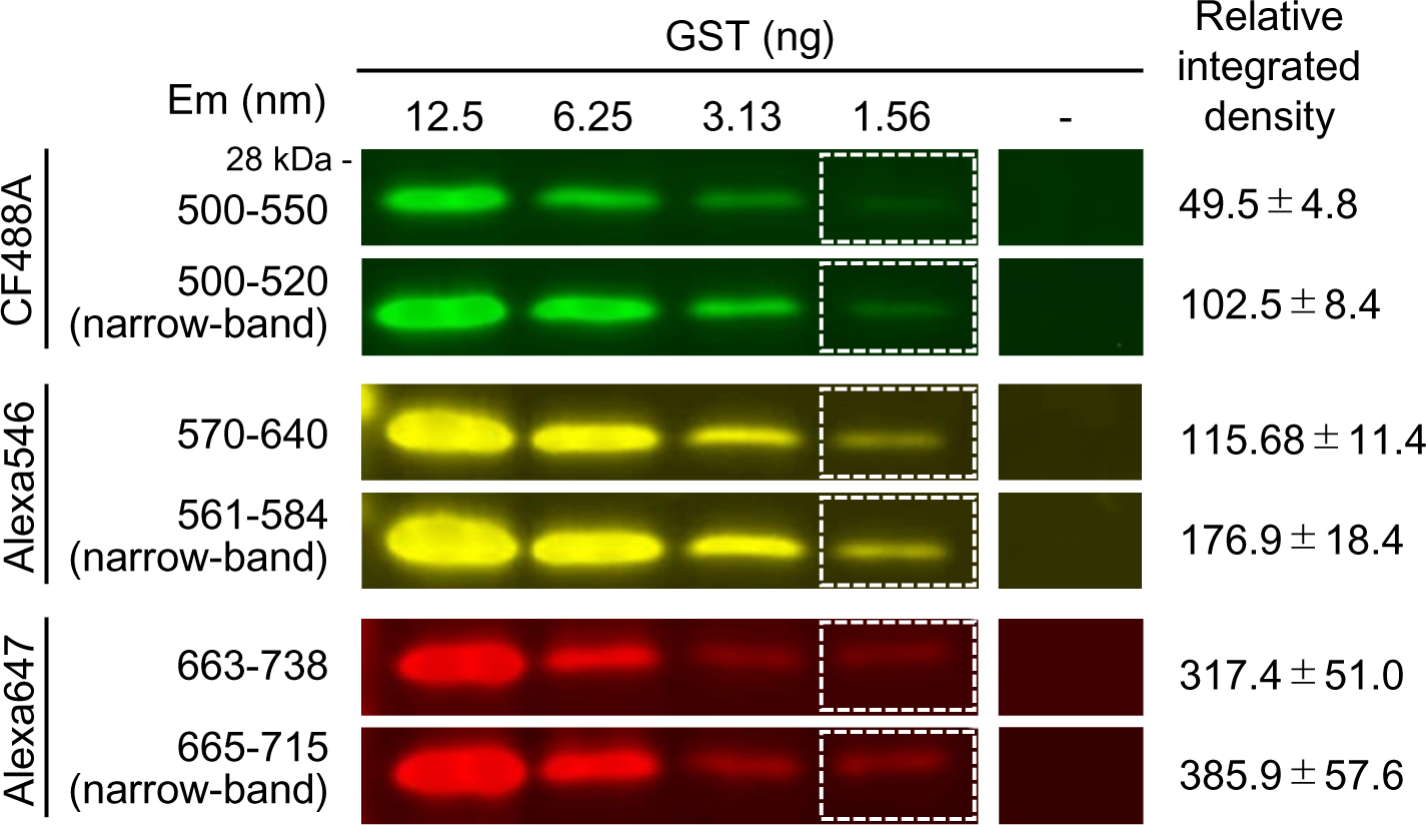
Reduction in noise signals by narrowing emission wavelength region. Effects of using narrow-band filters on detection sensitivity. Joined images of dilution series of GST and background area are shown. GST was visualized using anti-GST antibody and fluorescent dyes conjugated to secondary antibody (CF488A, Alexa 546, and Alexa 647). Emission wavelength (Em) of filters and relative integrated density of 1.56 ng GST using general filters (upper lane) and narrow-band filters (lower lane) are shown (mean ± standard error of three independent experiments). Exposure time was 1/3 seconds in the normal filter and 1/7.5 seconds in the narrow-band filter in detecting GFP and Alexa 546, and was 1–1.5 seconds in the normal filter and 1.5 seconds in the narrow-band filter in detecting Alexa 647.

To reduce the noise signals and enhance detection sensitivity, we used two approaches to optimize filters and fluorescent dyes. First, we optimized the filter to remove autofluorescence and separate it from excitation light. The excitation and emission filters used for capturing tissue images have wide transmission wavelength ranges to detect, signals from a wide wavelength range, but undesirable signals are also transmitted and can result in noise. Narrowing the transmission wavelength range of the filter can remove unnecessary signals to enhance detection sensitivity, but there is the risk of weakening the target signal intensity. Thus, we investigated whether noise was removed and the target signal intensity was maintained by using a filter with a narrower transmission wavelength range. We serially diluted recombinant GST, and labelled it with CF488A (green), Alexa 546 (yellow), and Alexa 647 (far-red). Each signal was detected using the general filter and the narrow-band filter, and the relative integrated density of GST was calculated. The relative integrated density of 1.56 ng GST is shown in Fig 2. In the green wavelength region (495–570 nm), the relative integrated density of GST was approximately doubled by using the narrow-band filter. The relative integrated density of GST was similarly enhanced in the yellow (570–590 nm) and far-red (710–850 nm) wavelength regions. These results indicated that removal of noise by narrowing the transmission wavelength range of the filter significantly enhanced the detection sensitivity.

### Use of high fluorescence intensity dyes to enhance detection sensitivity

**Fig 3:**
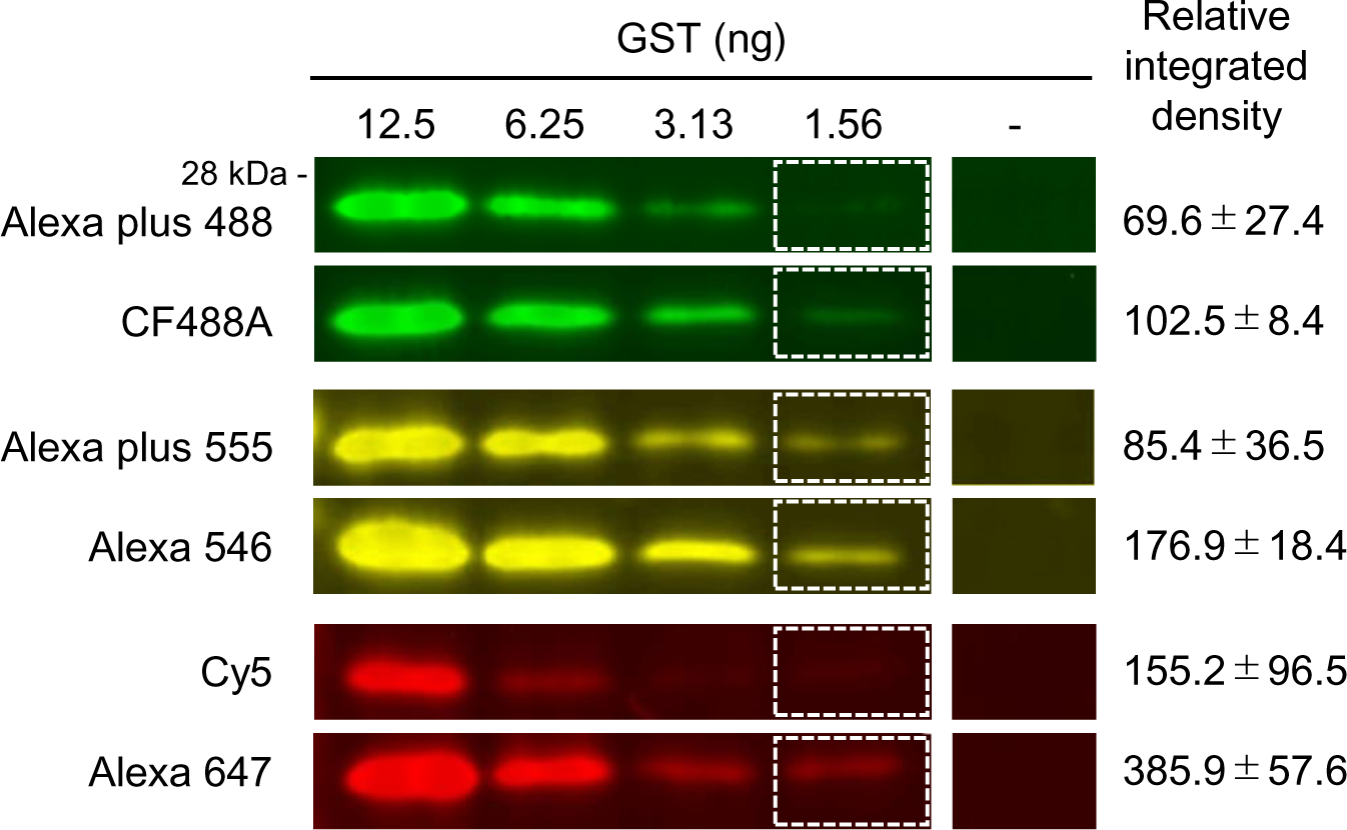
Increase in signal intensity by selecting appropriate fluorescent dyes. Comparison of detection sensitivity among fluorescent dyes. Joined images of dilution series of GST and background area are shown. GST was detected by anti-GST antibody, fluorescent dye conjugated to secondary antibody, and narrow-band filters (Em: 500–520 nm, 570–590 nm, and 665–715 nm). Relative integrated density of 1.56 ng GST signal is shown (mean ± standard error of three independent experiments). Exposure time was 1/1.5 seconds, 1/3 seconds and 1 second in green, yellow and far-red wavelength regions respectively.

Since the use of a narrow-band filter may decrease the target signal, we selected dyes with high fluorescence intensity to enhance the target signal. The fluorescence intensity is increased relative to the product of the molar extinction coefficient (ε) and fluorescence quantum yield (φ). To select fluorescent dyes that would enhance the signal, we compared the signal intensity of GST using dyes with different ε and φ in each wavelength range (Fig 3). In the green wavelength region, the relative integrated density of 1.56 ng GST was higher with CFF488A than with Alexa plus 488. In the yellow wavelength region, the relative integrated density of GST was higher with Alexa 546 than with Alexa plus 555, and in the far-red wavelength region, the relative integrated density of GST was higher with Alexa 647 than with Cy5. The product of the ε and φ of Cy5 is 75,000, and that of Alexa 647 is 89,100. This value may be used as an index to select a fluorescent dye, because those with higher values tended to show higher relative integrated density of GST in each wavelength region. These results confirmed that selection of an appropriate fluorescent dye enhanced the target signal and increased the detection sensitivity.

### Quantitative and simultaneous detection of multi-fluorescence signals

**Fig 4:**
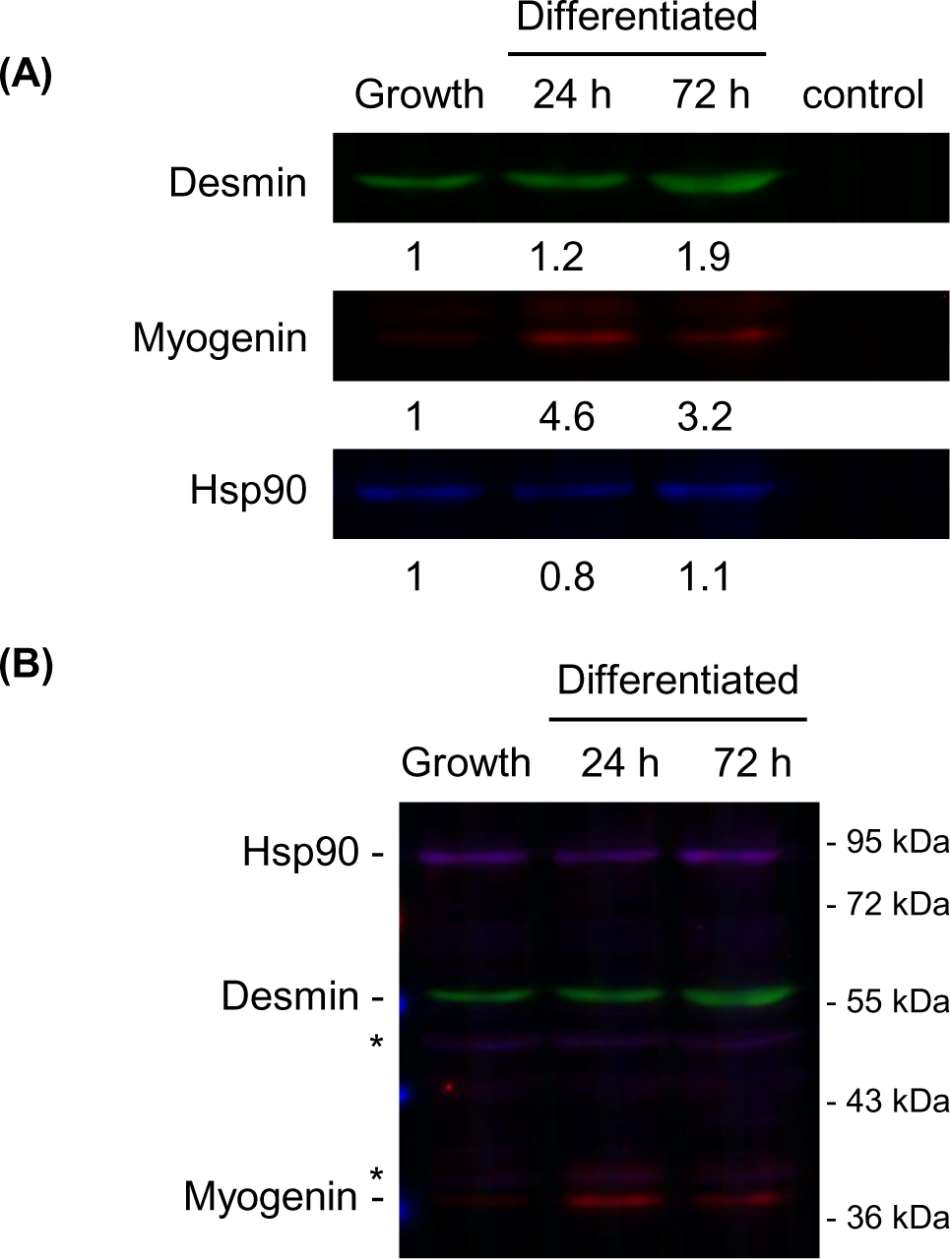
Practical application of the fluorescence detection method to analyze multiple proteins. (A) Joined images of fluorescent western blot of C2C12 myoblast cells under normal growth and differentiated conditions. Control lane contained sample buffer as negative control. Desmin (green), myogenin (red), and Hsp90 (blue) are displayed as false colors. Relative integrated density values normalized to the values at the growth condition are shown. Exposure time was 1/40 seconds in desmin, 1/8.5 seconds in myogenin and 1/5 seconds in Hsp90. (B) Merged images described in (A). Asterisk shows nonspecific band.

To confirm the practicality of merging fluorescence micrographs, we demonstrated the simultaneous detection of three proteins using optimized filters and fluorescent dyes (Table 1). With these optimized filter-dye combinations, the relative integrated density was linearly increased relative to amount of loaded GST (S1 Fig). Specifically, we detected desmin (muscle-specific intermediate filament), myogenin (muscle-specific transcription factor) and Hsp90 (molecular chaperone), which showed different expression patterns during skeletal muscle differentiation in C2C12 myoblast cells. Desmin expression increases upon induction of differentiation [8], myogenin is not expressed under normal growth conditions but increases after differentiation [9], and Hsp90 is expressed continuously [9]. Consistent with known expression patterns, the relative expression level of desmin was increased to 1.2 in 24 hours and to 1.9 in 72 hours after upon induction of differentiation. Myogenin was not expressed before differentiation, but its relative expression level increased to 4.6 in 24 hours and to 3.2 in 72 hours after differentiation, and Hsp90 was stably expressed. There was some antibody-derived noise in the detection of myogenin and Hsp90, but our results were consistent with those of previous reports (Fig 4). These results confirmed that multiple proteins could be detected concurrently and quantitatively by this fluorescence detection method using a fluorescence microscope.

**Table 1:** Optimized combinations of filter and fluorescence dye in each color wavelength region.

	Filter (Em)	Fluorescence dye
Green (495–570 nm)	Narrow-band (500–520 nm)	CF488A
Yellow (570–590 nm)	Narrow-band (561–584 nm)	Alexa546
Far-red (710–850 nm)	Narrow-band (665–715 nm)	Alexa647

Optimized combinations of filter and fluorescence dye in each color wavelength region (see Fig 4).

## Discussion

The main problems of fluorescence detection methods are low resolution and poor detection sensitivity. In this study, we successfully captured a high-resolution image by merging multiple images. This technique of capturing a wide-field image by merging fluorescence microscope images has been used to visualize immunostaining in various tissues. When merging images captured under the high-power objective lens, image distortion may occur because of objective lens aberration. However, images are captured under low-power objective lens in our method, and so there is little image distortion. We reduced noise and enhanced the signal intensity by using a narrow-band filter and selecting high fluorescence intensity dyes. Consequently, the detection sensitivity was enhanced to a level comparable to that of the chemiluminescence detection method. Therefore, our methods would be suitable for quantitative protein expression analysis with higher resolution and detection sensitivity. In future studies, both the filter and fluorescence dye should be further optimized to increase the color contrast in particular wavelength regions. As our method differs from the chemiluminescence method only in using fluorescent dyes conjugated to the secondary antibody and detecting signal using the fluorescence microscope, it enables fluorescence western blotting at a low installation cost without modifying the chemiluminescence method protocol.

Our method is not limited in the selection of target proteins, antibody host species, fluorescence dyes or filters, and does not require a special detector but only a standard fluorescence microscopy. One advantage of this fluorescent western blotting technique is that multiple proteins can be detected concurrently. Thus, we examined whether multiple proteins were detected using the optimized filter and fluorescent dyes. We detected three kinds of proteins simultaneously and quantitatively in the green, yellow, and far-red wavelength regions. Because the fluorescence microscope has a wide range of excitation wavelengths, we think that it is possible to detect up to five kinds of proteins concurrently by establishing appropriate conditions for detection in the blue and near-infrared wavelength regions. In addition, exposure time per vision is shorter than 1.5 seconds lessening the risk of the photo-bleaching of signals. However, because antibody-derived nonspecific bands may be detected depending on the type of target protein, the results may be complex and difficult to interpret. For exhaustive protein expression analyses, it is necessary to increase the number of useful wavelength regions. It would be useful to develop a technique that is not limited by the type of fluorescent dyes to analyze the expression of many proteins simultaneously.

## Supporting information

S1 Fig Correlations between the protein amount and relative integrated density. The relative integrated density values of GST using narrow-band filters (Em: 500–520 nm, 570–590 nm, and 665–715 nm) and fluorescent dyes (CF488A, Alexa 546, and Alexa 647, respectively) conjugated to the secondary antibody are shown (mean ± standard error of three independent experiments) described in Fig 3. The semi-linear increase slows down after a certain point, which might suggest that fluorescence signal starts to be saturated.
(TIF)
