# Supplementary figures and images for: Sensitive detection of fluorescence in western blotting by merging images
Source: PLoS One. 2018 Jan 19;13(1):e0191532. doi: 10.1371/journal.pone.0191532 (PMC5774814; doi:10.1371/journal.pone.0191532)

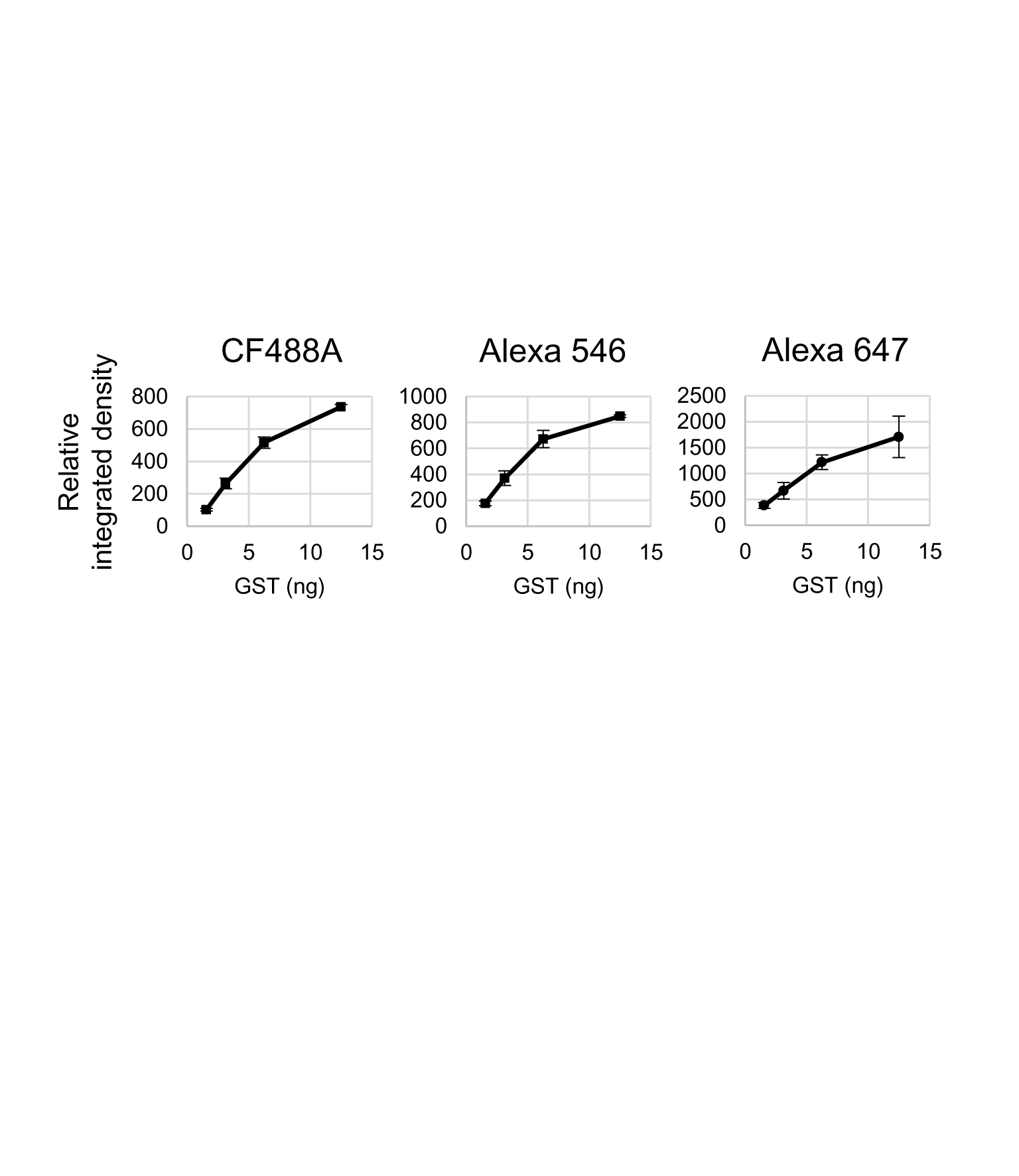

Supplement: S1 Fig — The relative integrated density values of GST using narrow-band filters (Em: 500–520 nm, 570–590 nm, and 665–715 nm) and fluorescent dyes (CF488A, Alexa 546, and Alexa 647, respectively) conjugated to the secondary antibody are shown (mean ± standard error of three independent experiments) described in Fig 3. The semi-linear increase slows down after a certain point, which might suggest that fluorescence signal starts to be saturated. (TIF) [file pone.0191532.s001.tif]
